# Oxygen dissociation from ferrous oxygenated human hemoglobin:haptoglobin complexes confirms that in the R-state α and β chains are functionally heterogeneous

**DOI:** 10.1038/s41598-019-43190-x

**Published:** 2019-05-01

**Authors:** Paolo Ascenzi, Fabio Polticelli, Massimiliano Coletta

**Affiliations:** 1 Interdepartmental Laboratory for Electron Microscopy, Roma Tre University, Via della Vasca Navale 79, I-00146 Roma, Italy; 2 Department of Sciences, Roma Tre University, Viale G. Marconi 446, I-00146 Roma, Italy; 3 National Institute of Nuclear Physics, Roma Tre Section, Via della Vasca Navale 84, I-00146 Roma, Italy; 4 Department of Clinical Sciences and Translational Medicine, University of Roma “Tor Vergata”, Via Montpellier 1, I-00133 Roma, Italy; 5 Interuniversity Consortium for the Research on the Chemistry of Metals in Biological Systems, Via Celso Ulpiani 27, I-70126 Bari, Italy

**Keywords:** Molecular medicine, Biophysical chemistry

## Abstract

The adverse effects of extra-erythrocytic hemoglobin (Hb) are counterbalanced by several plasma proteins devoted to facilitate the clearance of free heme and Hb. In particular, haptoglobin (Hp) traps the αβ dimers of Hb, which are delivered to the reticulo-endothelial system by CD163 receptor-mediated endocytosis. Since Hp:Hb complexes show heme-based reactivity, kinetics of O_2_ dissociation from the ferrous oxygenated human Hp1-1:Hb and Hp2-2:Hb complexes (Hp1-1:Hb(II)-O_2_ and Hp2-2:Hb(II)-O_2_, respectively) have been determined. O_2_ dissociation from Hp1-1:Hb(II)-O_2_ and Hp2-2:Hb(III)-O_2_ follows a biphasic process. The relative amplitude of the fast and slow phases ranges between 0.47 and 0.53 of the total amplitude, with values of *k*_off1_ (ranging between 25.6 ± 1.4 s^−1^ and 29.1 ± 1.3 s^−1^) being about twice faster than those of *k*_off2_ (ranging between 13.8 ± 1.6 s^−1^ and 16.1 ± 1.2 s^−1^). Values of *k*_off1_ and *k*_off2_ are essentially the same independently on whether O_2_ dissociation has been followed after addition of a dithionite solution or after O_2_ displacement by a CO solution in the presence of dithionite. They correspond to those reported for the dissociation of the first O_2_ molecule from tetrameric Hb(II)-O_2_, indicating that in the R-state α and β chains are functionally heterogeneous and the tetramer and the dimer behave identically. Accordingly, the structural conformation of the α and β chains of the Hb dimer bound to Hp corresponds to that of the subunits of the Hb tetramer in the R-state.

## Introduction

Hemoglobin (Hb), the most prominent intracellular circulating protein, is devoted to the O_2_ transport and the chemistry of reactive oxygen and nitrogen species^1–7^. Physiologically, Hb is released in plasma during the enucleation of erythroblasts and the hemolysis of senescent erythrocytes. Moreover, the intravascular release of Hb occurs during blood transfusion and represents a dramatic pathological complication of several diseases, including autoimmune, infectious, and inherited diseases^1,8–10^. The toxicity of extra-erythrocytic Hb depends on: (*i*) the heme-Fe-based production of free radicals, which induces the lipids peroxidation triggering the inflammatory cascade^11^, (*ii*) the sequestering of NO produced by endothelial cells lowering its bioavailability^12^, and (*iii*) the effects in the kidneys due to its renal filtration^13^.

The adverse effects of extra-erythrocytic Hb are counterbalanced by several plasma proteins devoted to facilitate the clearance of free heme and Hb. In particular, high and low density lipoproteins, albumin, and hemopexin ensure the complete clearance of the free heme, which is released into hepatic parenchymal cells by the CD91 receptor-mediated endocytosis of the hemopexin-heme complex. After conveying the heme intracellularly, hemopexin is released into the bloodstream and the heme is degraded^9,14–16^. Moreover, haptoglobin (Hp) is devoted to trap the extra-erythrocytic αβ dimers of Hb; the formation of Hb dimers, and in turn of the Hp:Hb complexes, is favored by the low extra-erythrocytic Hb concentration and its oxygenated state. By CD163 receptor-mediated endocytosis, the Hp:Hb complexes are delivered to the reticulo-endothelial system where they are degraded to release the heme. Heme-oxygenase catalyzes the heme conversion to biliverdin that is further transformed to bilirubin. The bilirubin is then exported from the macrophage and carried from albumin to the liver for conjugation in the hepatocytes and subsequent biliary excretion^9,15–22^.

In humans, two alleles of Hp (Hp1 and Hp2) are expressed as single polypeptide chains. In particular, Hp1 displays a single complement control protein (CCP) domain and a single serine protease-like (SP-like) domain, whereas Hp2 contains two CCP domains and one SP-like domain^23^. Both Hp1 and Hp2 are proteolytically cleaved into α and β chains, which are covalently linked by a disulfide bond(s). Hp1 and Hp2 alleles induce the formation of Hp1-1 dimers (covalently linked by Cys15 residues), Hp1-2 hetero-oligomers and Hp2-2 oligomers (covalently linked by Cys15 and Cys74 residues)^21^. The most abundant Hp2-2 species is the tetramer, but trimers and higher order oligomers have been reported^22–25^.

Each Hp β chain binds one αβ dimer of Hb, making extensive contacts with the Hb dimer-dimer interface^24,25^. Accordingly, values of (*i*) the dissociation equilibrium constants for the recognition of deoxygenated and oxygenated Hb (Hb(II) and Hb(II)-O_2_, respectively) dimers by Hp are 1 × l0^−7^ M and 1.3 × l0^−6^ M, respectively, and (*ii*) the second order rate constants for Hp:Hb complexation range between 5 × 10^5^ M^−1^ s^−1^ and 9 × 10^5^ M^−1^ s^−1^ ^26–28^. Since Hb(II)-O_2_ dissociates into αβ dimers preferentially with respect to Hb(II), the reaction of Hp with Hb represents a probe of the R-T transition of Hb^27,29^.

As the Hp:Hb complexes show functional properties similar to those of the Hb R-state, *e.g*. they display a high ligand specificity and show neither “heme-heme interactions” nor the Bohr effect^30–37^, we decided to investigate the kinetics of O_2_ dissociation from human Hb(II)-O_2_ dimers bound to human Hp phenotypes 1-1 and 2-2 (Hp1-1:Hb(II)-O_2_ and Hp2-2:Hb(II)-O_2_, respectively). The relevance of this approach is related to the fact that in this way it is possible to characterize the O_2_ dissociation from a pure population of α_1_β_1_ (and α_2_β_2_) dimers without any interference from tetrameric species. Therefore, it is possible to sort out the contribution of the α_1_β_1_ (and α_2_β_2_) inter-subunit contacts from the α_1_β_2_ (and α_2_β_1_) ones, which are destroyed upon dimerization^38^. O_2_ dissociation from Hp1-1:Hb(II)-O_2_ and Hp2-2:Hb(II)-O_2_ follows a biphasic process, the fast process (*i.e*., *k*_off1_ values) being about 2-fold faster than the slow one (*i.e*., *k*_off2_ values). Values of *k*_off1_ and *k*_off2_ are similar to those for the deoxygenation of isolated α(II)-O_2_ and β(II)-O_2_ chains of Hb^39,40^ and identical to those for the dissociation of the first O_2_ molecule from tetrameric Hb(II)-O_2_^41^. The close similarity for the observed heterogeneity between experiments of O_2_ replacement by CO and those of O_2_ dissociation by sodium dithionite allows to state unequivocally for the first time that the biphasicity cannot be referable to a negative cooperativity in the α_1_β_1_ (and α_2_β_2_) dimers. It clearly demonstrates that the α and β chains of the oxygenated R-state of Hb are functionally heterogeneous to the same extent both in the tetrameric and in the dimeric assembly. Accordingly, the conformation of the α and β chains of the Hb dimer bound to Hp corresponds to that of the α_1_β_1_ (and α_2_β_2_) dimers in the R-state tetramer^24,25,42,43^.

## Materials

Human Hp1-1 and Hp2-2 were purchased from Athens Research & Technology, Inc. (Athens, GA, USA). Human oxygenated Hb was prepared as previously reported^44^. The oxygenated Hp:Hb complexes were prepared by mixing oxygenated Hb with Hp1-1 and Hp2-2 at pH 7.0 and 20.0 °C^29^. The dimeric Hp:tetrameric Hb stoichiometry was 1:1. To avoid the occurrence of free Hb, a 20% excess of Hp1-1 and Hp2-2 was present in all samples. The absence of free Hb was checked by gel electrophoresis^31^.

CO was purchased from Linde AG (Höllriegelskreuth, Germany). The CO solution was prepared by keeping in a closed vessel the 5.0 × 10^−2^ M phosphate buffer solution (pH = 7.0) under CO at *P* = 760.0 mm Hg anaerobically (*T* = 20.0 °C). The solubility of CO in the aqueous buffered solution is 1.03 × 10^−3^ M, at *P* = 760.0 mm Hg and *T* = 20.0 °C^44^.

All the other chemicals were purchased from Sigma-Aldrich (St. Louis, MO, USA). All chemicals were of analytical grade and were used without further purification.

## Methods

Kinetics of O_2_ dissociation from Hp1-1:Hb(II)-O_2_ and Hp2-2:Hb(II)-O_2_ (final concentration, 3.2 × 10^−6^ M to 5.5 × 10^−6^ M) were investigated either by mixing oxygenated Hp:Hb(II) complexes with a dithionite solution (final concentration, 1.0 × 10^−3^ M)^33^ or by O_2_ replacement with CO in the presence of dithionite (final concentrations, 5.0 × 10^−4^ M and 3.0 × 10^−3^ M, respectively)^41^. No gaseous phase was present.

**Figure 1:**
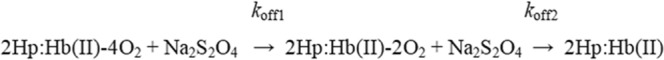
Dithionite-induced O_2_ dissociation from Hp1-1:Hb(II)-O_2_ and Hp2-2:Hb(II)-O_2_.

**Figure 2:**
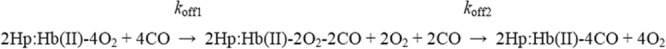
O_2_ replacement by CO in Hp1-1:Hb(II)-O_2_ and Hp2-2:Hb(II)-O_2_.

Since with both methods, the O_2_ dissociation time courses from Hp1-1:Hb(II)-O_2_ and Hp2-2:Hb(II)-O_2_ display two exponentials, they have been analyzed in the framework of either Fig. 1 or Fig. 2.

Values of the apparent first-order rate constants for O_2_ dissociation from Hp1-1:Hb(II)-O_2_ and Hp2-2:Hb(II)-O_2_ (*i.e*., *k*_off1_ and *k*_off2_) were obtained according to Eqs 1 and 2^45^:

1 $$\begin{array}{rcl}{[{\rm{Hp}}:{\rm{Hb}}({\rm{II}})-{{\rm{O}}}_{2}]}_{{\rm{t}}} & = & a\times {[{\rm{Hp}}:{\rm{Hb}}({\rm{II}})-{{\rm{O}}}_{2}]}_{{\rm{i}}}\times {{\rm{e}}}^{-k{\rm{off}}1\times t}\\  &  & \,\,\,+\,b\times {[{\rm{Hp}}:{\rm{Hb}}({\rm{II}})-{{\rm{O}}}_{2}]}_{{\rm{i}}}\times {{\rm{e}}}^{-k{\rm{off}}2\times t}\end{array}$$

2 $$\begin{array}{rcl}{[{\rm{Hp}}:{\rm{Hb}}({\rm{II}})-{{\rm{O}}}_{2}]}_{{\rm{t}}} & = & a\times {[{\rm{Hp}}:{\rm{Hb}}({\rm{II}})-{{\rm{O}}}_{2}]}_{{\rm{i}}}\times (1-{{\rm{e}}}^{-k{\rm{off}}1\times t})\\  &  & \,\,\,+\,b\times {[{\rm{Hp}}:{\rm{Hb}}({\rm{II}})-{{\rm{O}}}_{2}]}_{{\rm{i}}}\times (1-{{\rm{e}}}^{-k{\rm{off}}2\times t})\end{array}$$

depending on the observation wavelength. Hp:Hb(II)-O_2_ indicates either Hp1-1:Hb(III)-O_2_ or Hp2-2:Hb(III)-O_2_, and *a* and *b* indicate the relative amplitude of the fast and slow binding process, respectively (*i.e*., *a* + *b* = 1; *i.e*., 100%).

O_2_ dissociation from Hp1-1:Hb(II)-O_2_ and Hp2-2:Hb(II)-O_2_ was investigated at pH 7.0 (5.0 × 10^−2^ phosphate buffer) and 20.0 °C. Kinetics was monitored by single-wavelength stopped-flow spectroscopy between 380 and 460 nm. The amplitude of the time courses for conversion of Hp:Hb(II)-O_2_ to Hp:Hb(II)-CO and of Hp:Hb(II)-O_2_ to Hp:Hb(II) was normalized to 420 and 430 nm, respectively. All kinetic experiments have been carried out with the BioLogic SFM-200 rapid-mixing stopped-flow apparatus (Claix, France); the dead-time of the stopped-flow apparatus was 1.4 ms and the observation chamber was 1 cm.

The results (from at least four experiments) are given as mean values plus or minus the corresponding standard deviation. All data were analyzed using the GraphPad Prism program, version 5.03 (GraphPad Software, La Jolla, CA, USA).

Comparison of the three-dimensional structure of the αβ dimer of Hb bound to Hp (PDB code 5JDO; resolution 3.2 Å)^43^ with the corresponding subunits of the Hb tetramer in the R-state (PDB code 2DN1; resolution 1.25 Å)^42^, including calculation of root mean square deviation (rmsd) values, has been carried out using SwissPDBViewer^46^.

## Results and Discussion

**Figure 3:**
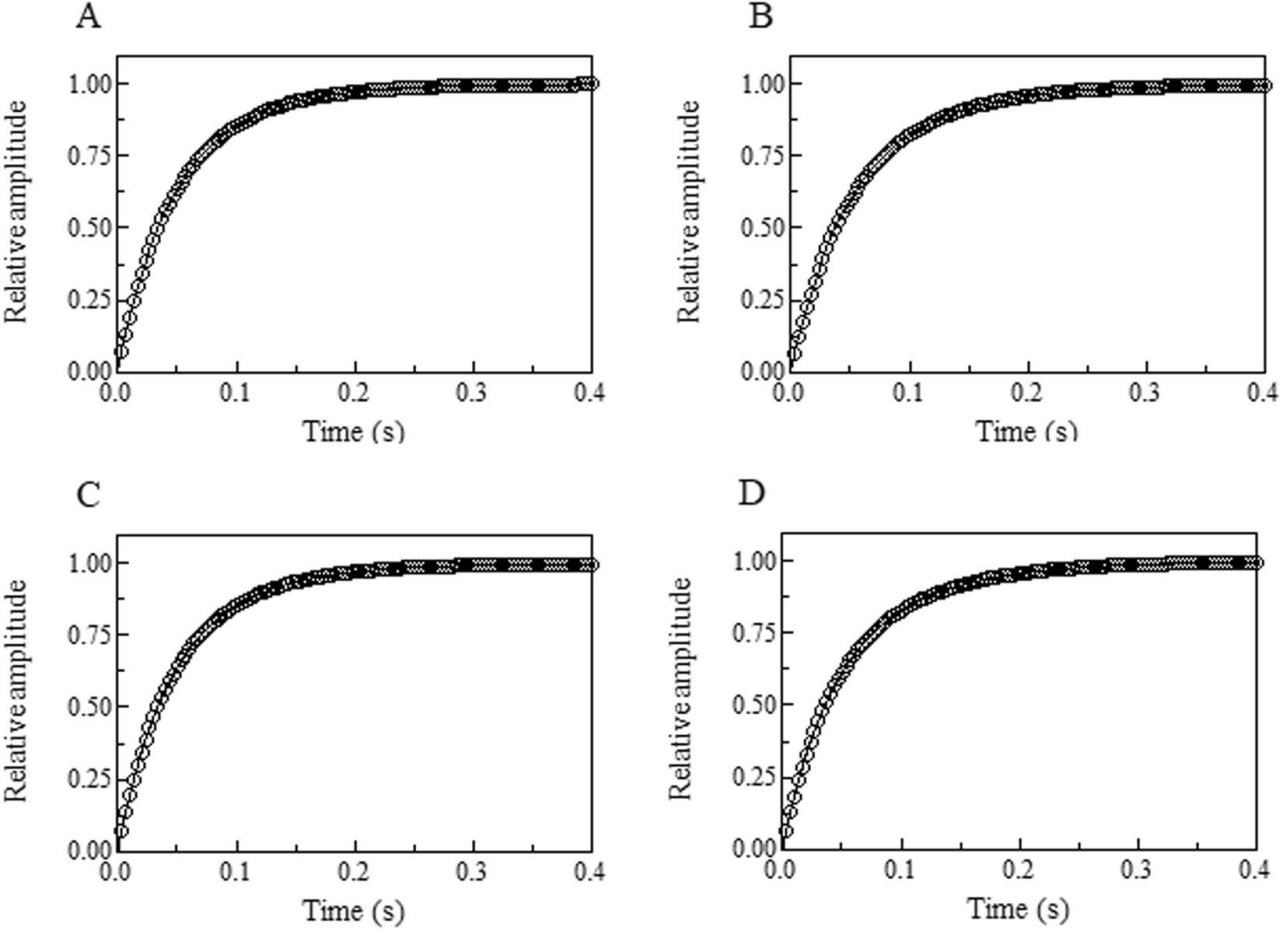
Average time course of O_2_ dissociation from Hp1-1:Hb(II)-O_2_ (panels A and C) and from Hp2-2:Hb(II)-O_2_ (panels B and D) by mixing the Hp-:Hb(II)-O_2_ solutions with the CO solution in the presence of dithionite (panels A and B, respectively) and with the dithionite solution only (panels C and D). The continuous lines were calculated according to Eq. 2 with the following parameters: (panel A) *a* = 0.49, *k*_off1_ = 27.4 s^−1^, *b* = 0.51, and *k*_off2_ = 15.1 s^−1^; (panel B) *a* = 0.47, *k*_off1_ = 26.3 s^−1^, *b* = 0.53, and *k*_off2_ = 13.1 s^−1^; (panel C) *a* = 0.52, *k*_off1_ = 28.3 s^−1^, *b* = 0.48, and *k*_off2_ = 14.2 s^−1^; and (panel D) *a* = 0.49, *k*_off1_ = 28.8 s^−1^, *b* = 0.51, and *k*_off2_ = 12.6 s^−1^.

Under all the experimental conditions, O_2_ dissociation from Hp1-1:Hb(II)-O_2_ and Hp2-2:Hb(III)-O_2_ follows a biphasic process upon mixing the Hp1-1:Hb(II)-O_2_ and Hp2-2:Hb(III)-O_2_ solutions either with the dithionite solution or the CO solution in the presence of dithionite (Fig. 3). This is supported by statistical analysis (Table 1) and the residual distribution shown in Fig. 3 of the Supplementary Information section.

**Table 1:** Values of *k*_off_ for O_2_ dissociation from Hp:Hb(II)-O_2_ complexes.

Heme-protein	Method	*k*_off1_ (s^−1^)	*k*_off2_ (s^−1^)
Hp1-1:Hb^a,#^	O_2_ replacement from CO	29.1 ± 1.3	16.1 ± 1.2
	O_2_ consumption by dithionite	27.6 ± 2.2	15.1 ± 1.6
Hp2-2:Hb^a,#^	O_2_ replacement from CO	25.6 ± 1.4	13.8 ± 1.6
	O_2_ consumption by dithionite	27.8 ± 1.6	14.7 ± 0.9
Hp2-2:Hb^b^	O_2_ consumption by dithionite	59.5 ± 5.5	11 ± 3
Hb	O_2_ replacement from CO	21.2 ± 2.6^c^	13.0 ± 1.4^c^
α-Chains	O_2_ replacement from CO^d^	28 ± 6	—
	O_2_ consumption by dithionite^d^		
	O_2_ replacement from CO^e^	28 ± 3	—
β-Chains	O_2_ replacement from CO^d^	—	16 ± 5
	O_2_ consumption by dithionite^d^		
	O_2_ replacement from CO^e^	—	18 ± 2

^a^pH 7.0 and 20.0 °C. Present study.

^#^*k*_off1_ *versus k*_off2_ Student’s *t*-test, *p* < 0.0001. Present study.

^b^pH 7.0 and 20.0 °C^33^. Since errors of *k*_off1_ and *k*_off2_ values are not available, errors have been calculated arbitrarily as the average ± the reported interval, for the homogeneous comparison.

^c^Dissociation of the first O_2_ molecule from Hb(II)-O_2_. pH 7.0 and 20.0 °C^41^.

^d^pH 7.0 and 20.0 °C^39^.

^e^pH 7.0 and 20.0 °C^40^.

**Figure 4:**
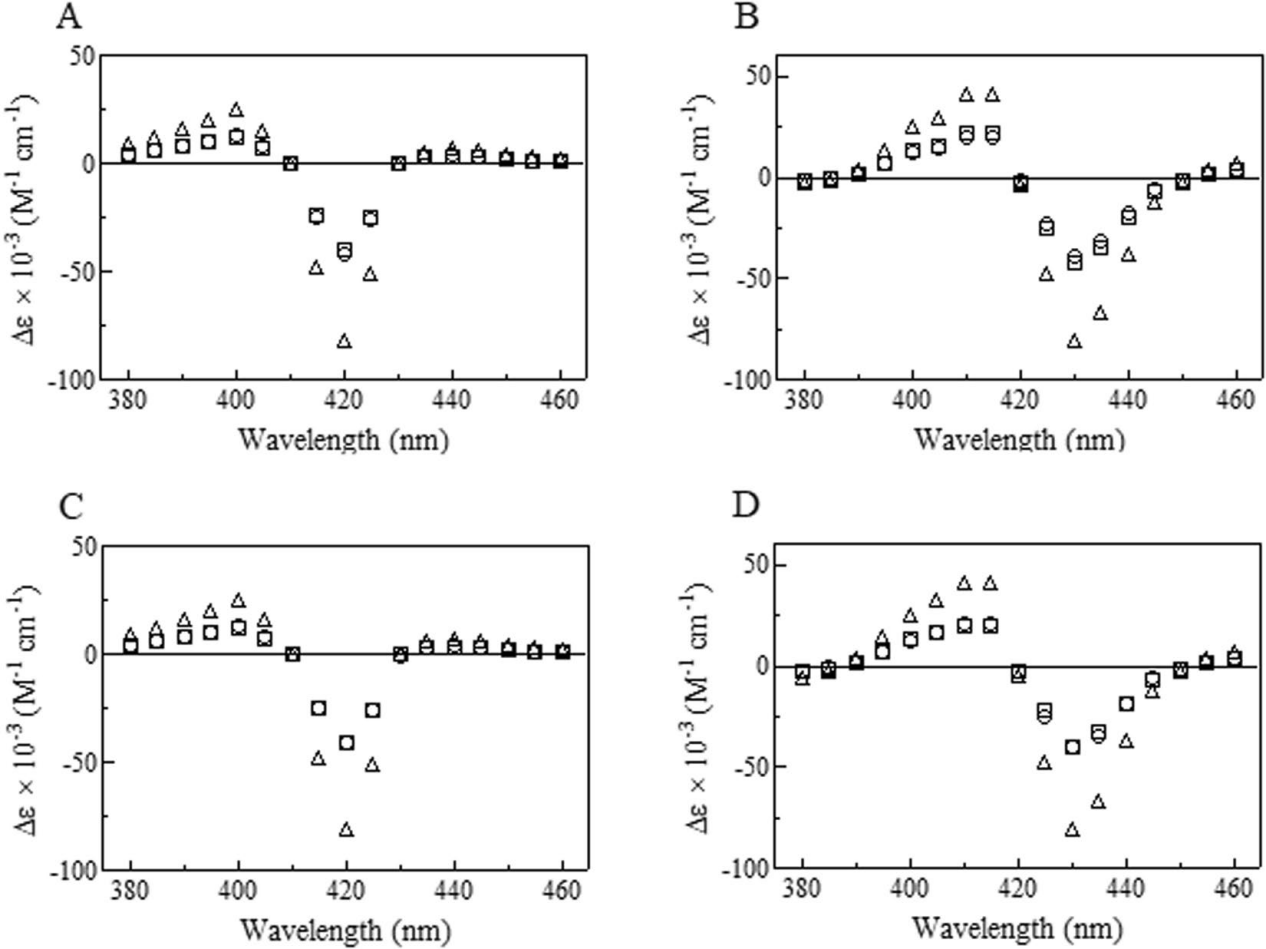
Difference absorbance spectra of the fast (circles) and the slow (squares) phases of Hp1-1:Hb(II)-O_2_ *minus* Hp1-1:Hb(II)-CO (panel A), Hp1-1:Hb(II)-O_2_ *minus* Hp1-1:Hb(II) (panel B), Hp2-2:Hb(II)-O_2_ *minus* Hp2-2:Hb(II)-CO (Panel C), and Hp2-2:Hb(II)-O_2_ *minus* Hp2-2:Hb(II) (panel D). Triangles indicate the total difference absorbance spectra. The relative amplitude of the fast and slow phases ranges between 0.47 and 0.53 over the whole wavelength range explored (*i.e*., between 380 and 460 nm).

The relative amplitude of the fast and slow phases (*i.e*., values of *a* and *b*, respectively; see Eqs 1 and 2) ranges between 0.47 and 0.53 of the total amplitude (*i.e*., *a* + *b* = 1) over the whole wavelength range explored (*i.e*., between 380 and 460 nm). Moreover, the difference absorbance spectra of the fast and the slow phases of Hp1-1:Hb(II)-O_2_ *minus* Hp1-1:Hb(II)-CO overlap with those of Hp2-2:Hb(II)-O_2_ *minus* Hp2-2:Hb(II)-CO and the difference absorbance spectra of the fast and the slow phases of Hp1-1:Hb(II)-O_2_ *minus* Hp1-1:Hb(II) are superimposable to those of Hp2-2:Hb(II) *minus* Hp2-2:Hb(II)-CO. In turn, they display the same shape of the overall difference absorbance spectra of Hp1-1:Hb(II)-O_2_ *minus* Hp1-1:Hb(II)-CO and Hp2-2:Hb(II)-O_2_ *minus* Hp2-2:Hb(II)-CO, and of Hp1-1:Hb(II)-O_2_ *minus* Hp1-1:Hb(II) and Hp2-2:Hb(II)-O_2_ *minus* Hp2-2:Hb(II) (Fig. 4). The fast process of Hp1-1:Hb(II)-O_2_ and Hp2-2:Hb(II)-O_2_ deoxygenation is about 2-fold faster than the slow one with values of *k*_off1_ ranging between 25.6 ± 1.4 s^−1^ and 29.1 ± 1.3 s^−1^ and *k*_off2_ ranging between 13.8 ± 1.6 s^−1^ and 16.1 ± 1.2 s^−1^. Moreover, values of *k*_off1_ and *k*_off2_ are closely similar for the Hp1-1:Hb(II)-O_2_ and Hp2-2:Hb(II)-O_2_ species (Table 1).

Interestingly, values of *k*_off1_ and *k*_off2_ are closely similar to both those for the deoxygenation of isolated α(II)-O_2_ and β(II)-O_2_ chains of Hb (28 ± 6 s^−1^ and 16 ± 5 s^−1^, respectively^39^; and 28 ± 3 s^−1^ and 18 ± 2 s^−1^, respectively^40^) and those for the dissociation of the first O_2_ molecule from tetrameric Hb(II)-O_2_ (21.2 ± 2.6 s^−1^ and 13.0 ± 1.4 s^−1^, respectively)^41^ (Table 1). Of note, while for isolated chains the O_2_ dissociation rate constant is faster for the α-chains^39^, in the case of tetrameric Hb(II)-O_2_ the dissociation of the first O_2_ molecule from the tetra-ligated species seems to be faster for the β-subunit^41^. This assembly-linked subunit functional difference has been suggested to reflect a structural change(s) of the α-chain upon the tetrameric assembly leading to a slower O_2_ dissociation rate constant. This finding has been also confirmed by subsequent observations on the kinetics of O_2_ displacement from oxygenated Hb by CO^47,48^, where the subunit characterized by the faster phase displays a higher affinity for organic phosphate, which is known to bind at the β-dyad axis^49,50^. Therefore, it seems very reasonable to identify the β-subunit as the one characterized by the faster O_2_ dissociation rate in the tetrameric R-state of Hb.

In the case of the Hp:Hb(II)-O_2_ complexes, a different situation occurs since Hp binds the α_1_β_1_ (and the α_2_β_2_) dimers^24,25^; therefore, parameters here reported (Table 1) reflect the functional features of this dimeric structure. In a previous paper on the O_2_ binding properties of the Hp2-2:Hb(II) complex^33^ the biphasic O_2_ dissociation process was detected, but a two-fold higher rate was reported for the fast phase (*i.e*., 59.5 ± 5.5 s^−1^) whereas the slower rate is closely similar to what observed by us (Table 1). At the moment, we have no obvious explanation for this discrepancy between data here described and previous ones^33^, but indeed the close similarity for O_2_ dissociation rates by both (*i*) mixing Hp:Hb(II)-O_2_ complexes with dithionite (thus fully deoxygenating Hp:Hb(II)-O_2_ to Hp:Hb(II), see Fig. 1) and (*ii*) displacing O_2_ with CO (thus keeping always a fully liganded form, see Fig. 2), as observed by us (Table 1), rules out the possibility of a negative cooperativity within the α_1_β_1_ dimer, definitely assigning the biphasicity to a different kinetic behavior of the two subunits.

Further, data shown in Table 1 confirm the view that: (*i*) the αβ dimers of Hb(II)-O_2_ bound to Hp1-1 and Hp2-2 are in the R-state as reported for isolated α(II)-O_2_ and β(II)-O_2_ chains and (*ii*) Hp1-1 and Hp2-2 species affect to the same extent the O_2_ dissociation from Hp1-1:Hb(II)-O_2_ and Hp2-2:Hb(III)-O_2_. In fact, the two CCP domains present in each Hp monomer are involved in the protein dimerization and do not participate to the recognition of the αβ dimers of Hb(II)-O_2_^23–25^.

**Figure 5:**
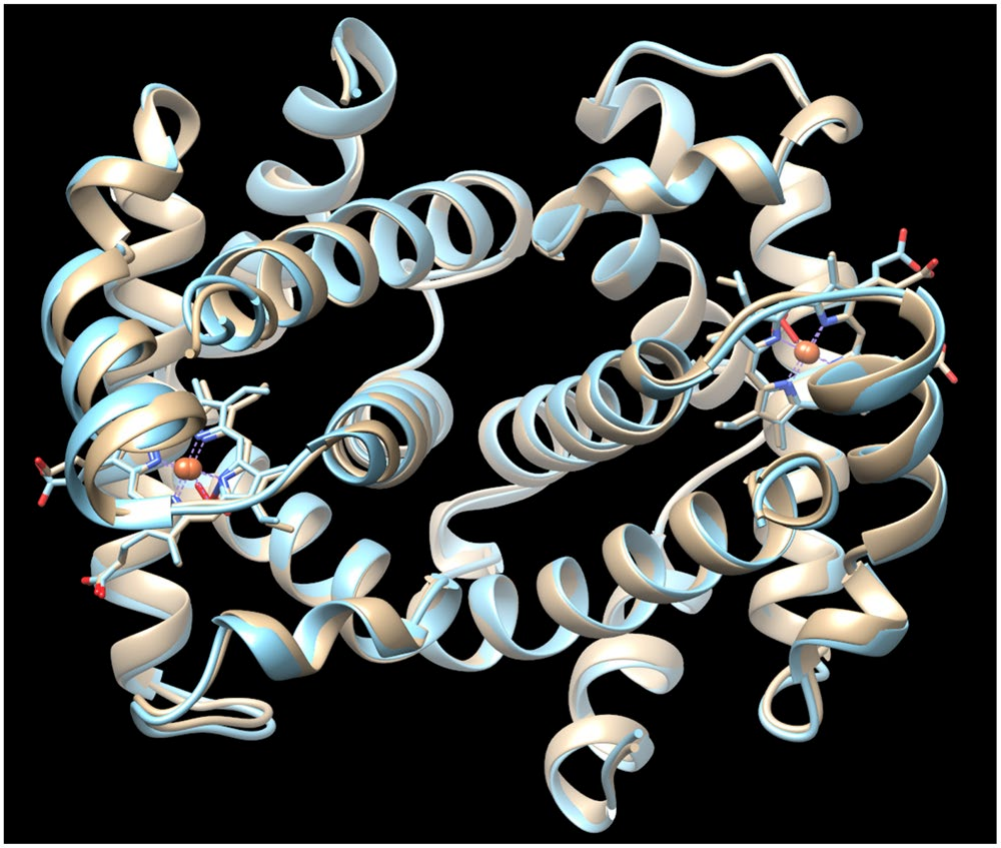
Structural superposition of the three-dimensional structure of the αβ dimer of Hb bound to Hp (cyan; PDB code 5JDO)^43^ to the corresponding subunits of the Hb tetramer in the R-state (light brown; PDB code 2DN1)^42^. The heme-Fe moieties are shown in stick representation. Iron atoms are represented by orange spheres and O_2_ molecules as red sticks. The figure has been drawn using the UCSF Chimera software^52^.

Lastly, the similar reactivity of the αβ dimers of Hb(II)-O_2_ bound to Hp and of the tetrameric R-state is in keeping with structural data. Indeed, both subunits of the αβ dimers of Hb(II)-O_2_ bound to Hp^43^ display a conformation superimposable to that of the α and β chains of tetrameric oxygenated Hb with the Fe(II) atom positioned in the plane of the heme group^42^ (Fig. 5). This is confirmed by the very low backbone rmsd value (0.56 Å) calculated upon best fitting the three-dimensional structure of the αβ dimer of Hb bound to Hp (PDB code 5JDO)^43^ to the corresponding subunits of the Hb tetramer in the R-state (PDB code 2DN1)^42^.

A final consideration is required with respect to the consequences of these data for the comprehension of the cooperative mechanism of O_2_ binding to Hb. It has been shown that the ligand-linked quaternary structural change, responsible for the cooperativity, is connected to a shift of the α_1_β_2_ (as well as of α_2_β_1_) interfaces^2,51^, as also suggested by the fact that at very high ionic strength the dissociation into α_1_β_1_ (and α_2_β_2_) dimers brings about the disappearance of cooperativity^38^, indicating that dimers are in a R-like quaternary state. However, the evidence of a functional heterogeneity for the O_2_ dissociation in the R state^41,48^ raised the question on the role of the α_1_β_1_ (and α_2_β_2_) subunit interaction in the energetics of cooperativity. Although the stabilization of the dimeric assembly upon interaction of Hb with Hp has been established since long time^27^ and a kinetic heterogeneity for the O_2_ dissociation has been shown also for the Hp:Hb complex^33^, it is only the close similarity, reported in this paper for the first time, between the O_2_ dissociation rates measured by full deoxygenation and O_2_ displacement by CO (Fig. 3) that allows to state that no ligand-linked structural change occurs at the α_1_β_1_ (and α_2_β_2_) subunit interaction surface, as also supported by the substantial identity for the dimer structure in the Hp:Hb complex and in the tetrameric oxygenated Hb (Fig. 5).

## Supplementary information

Figure 1 SI
