# Supplementary material for: Oxygen dissociation from ferrous oxygenated human hemoglobin:haptoglobin complexes confirms that in the R-state α and β chains are functionally heterogeneous
Source: Sci Rep. 2019 May 1;9:6780. doi: 10.1038/s41598-019-43190-x (PMC6494993; doi:10.1038/s41598-019-43190-x)
Supplement: Supplementary file 1 — Figure 1 SI [file 41598_2019_43190_MOESM1_ESM.pdf]

Supplementary Information

Oxygen dissociation from ferrous oxygenated human hemoglobin:haptoglobin complexes confirms that in the R-state α and β chains are functionally heterogeneous [¶]

Paolo Ascenzi, [a,*] Fabio Polticelli, [b,c] and Massimiliano Coletta [d,e]

[a] Interdepartmental Laboratory for Electron Microscopy,
Roma Tre University, Via della Vasca Navale 79, I-00146 Roma, Italy

[b] Department of Sciences, Roma Tre University, Viale G. Marconi 446, I-00146 Roma, Italy

[c] National Institute of Nuclear Physics, Roma Tre Section, Via della Vasca Navale 84, I-00146 Roma, Italy

[d] Department of Clinical Sciences and Translational Medicine, University of Roma "Tor Vergata", Via Montpellier 1, I-00133 Roma, Italy

[e] Interuniversity Consortium for the Research on the Chemistry of Metals in Biological Systems, Via Celso Ulpiani 27, I-70126 Bari, Italy

Running title: O_2 dissociation from oxygenated human haptoglobin:hemoglobin complexes

Keywords: Human haptoglobin 1-1; human haptoglobin 2-2; human hemoglobin; human haptoglobin 1-1:hemoglobin complex; human haptoglobin 2-2:hemoglobin complex; O_2 dissociation; kinetics.

Abbreviations: Hp1-1:Hb(II)-O_2, ferrous oxygenated Hp1-1:Hb complex; Hp2-2:Hb(II)-O_2, ferrous oxygenated Hp2-2:Hb complex.

* Corresponding author: Prof. Paolo Ascenzi, Interdepartmental Laboratory for Electron Microscopy, Roma Tre University, Via della Vasca Navale 79, I-00146 Roma, Italy.
Tel.: +39-06-5733-3621; fax: +39-06-5733-6321; e-mail address: ascenzi@uniroma3.it (P. Ascenzi).

[¶] This paper is dedicated to Prof. Emilia Chiancone, deceased on December 18th 2018, who pioneered structure-function relationships of high molecular weight heme-proteins.

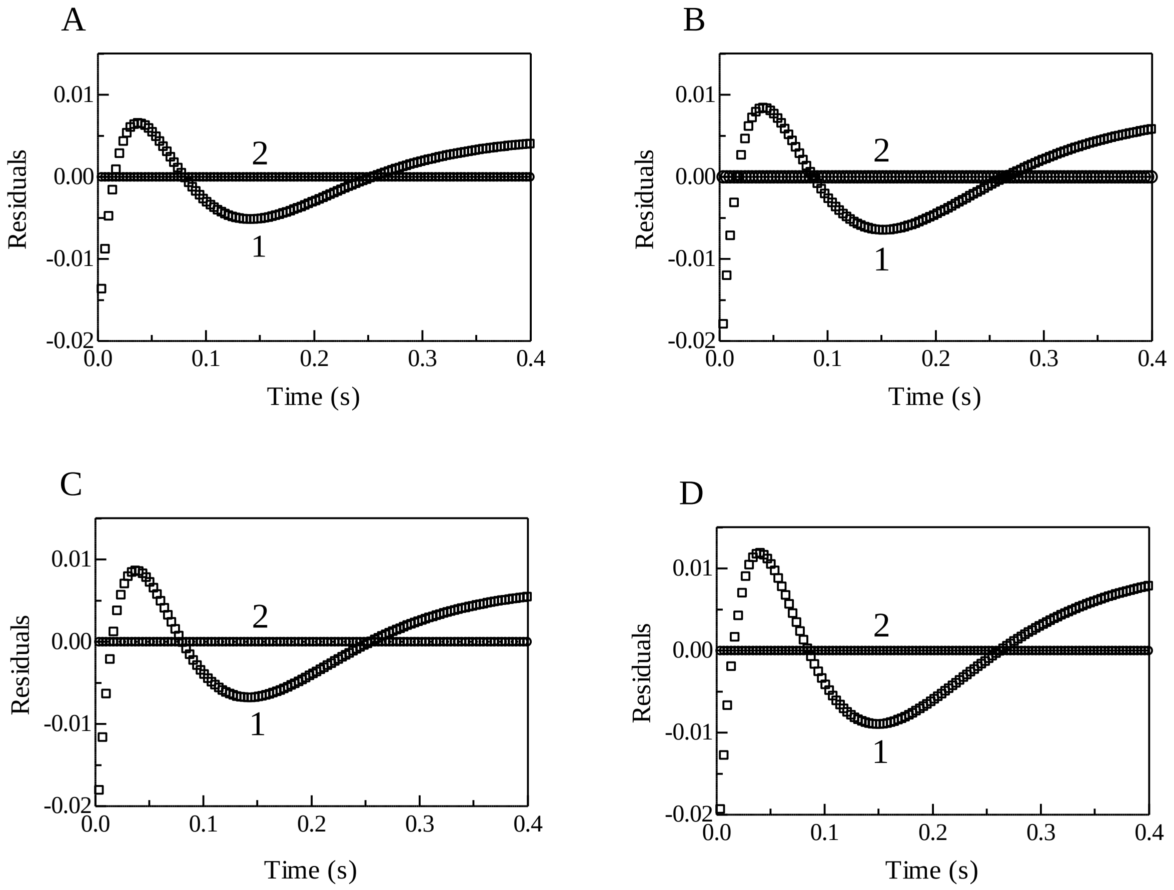

Figure 1 SI. Residuals obtained from the non-linear least-squares fitting with one (1) and two (2) exponential(s) of data referring to O_2 dissociation from Hp1-1:Hb(II)-O_2 (panels A and C) and from Hp2-2:Hb(II)-O_2 (panels B and D) by mixing the Hp1-1:Hb(II)-O_2 and Hp2-2:Hb(II)-O_2 solutions with the CO solution in the presence of dithionite (panels A and B, respectively) and with the dithionite solution only (panels C and D). All the fittings refer to data reported in Figure 1.
